# Obstetrics

**Published:** 1859-09

**Authors:** 


### OBSTETRICS.

1. Puerperal Convulsions successfully treated by Croton Oil Suppositories. *Letter from Mr. Overton.*—Puerperal convulsions are usually so alarmingly dangerous, and cause so much anxiety, both to relatives and friends—ay, and to the medical attendant, at the same time—and are often so rebellious to all treatment, that I trust you will deem the two following cases, extracted from my note-book, worthy of insertion in your columns.

Case 1.—Mrs. R., aged twenty-four, married about two years. A miscarriage took place six months after marriage, when she suffered considerably from nervous excitement, which, however, soon yielded to appropriate treatment. She was again pregnant in September, 1858, and enjoyed good health till February, 1859, when I received a summons to visit her immediately. I found her suffering from general anasarca, with excruciating pain in the head, restlessness, vomiting, and febrile symptoms. Upon the whole, I did not like the appearance of those symptoms. I prescribed saline aperients, with alteratives, etc.; cold applications to the head. The symptoms did not yield to this treatment: constipation gave some trouble.

March 15.—I was again sent for in great haste; the husband stating that his wife was in a fit, and all feared she was dying. I was soon with her, and found her suffering from epileptiform puerperal convulsions, frothy blood issuing from her mouth, and there were clonic spasms present. I at once abstracted blood from the arm, to about thirty ounces, put six to eight grains of calomel on her tongue, administered an enema of senna, etc., and applied a blister to the nape of the neck. These, combined with cold applications, produced little or no other effect than that of mitigating the acute pain in the head, between the convulsive paroxysms, which paroxysms became frequent. The patient having had no relief from the bowels, I deemed it advisable to mix six drops of croton oil with lard, in the form of a suppository, and so use it. This produced tenesmus, with a sense of smarting, burning heat in the lower bowel; but relief followed, and a copious watery evacuation almost immediately ensued. The patient had no more convulsions. Upon examination, I found no signs of on-coming labor, nor did the slightest chance of producing it present itself.

16th.—On my visit this morning, I found Mrs. R. calm, and free from convulsive symptoms; the anasarca had disappeared; micturition normal; bowels relieved (largely) four times; os uteri in the same state as before.

17th.—I found her dressed, and sitting by the fire in a lower room.

24th.—I was again sent for; and this time I found her in labor, the os uteri fully dilated, and the head presenting. Fearing a recurrence of the convulsions, I turned the child, and delivered her of a still-born, putrid child.

25th.—I found her quiet and comfortable, she had passed a good night, and from that period she went on well without one unfavorable symptom.

Case 2.—*Puerperal Convulsions; recovery; subsequent death from swallowing a row of front false teeth.* February 18.—Anna F., aged twenty-two, a country servant, primipara, unmarried, six months advanced in pregnancy. The man to whom she was engaged to be married left the neighborhood, and married another woman. This event produced great despondency, and on her way home to see her mother, she was seized with convulsions about every ten minutes. At this time she lost four front teeth, fixed on a silver plate; they could not be found. She had swallowed them. I was sent for, and found her suffering from puerperal convulsions. Blood issued from her mouth; the tongue bitten severely; clonic spasms; head cool; pulse small and compressible; micturition scanty; bowels not open. Upon examination, I found the os uteri high up in the pelvis contracted. I introduced the catheter, and some urine came away. I then administered an enema of castor oil and ol. terebinth, and put 10 grs. of calomel on her tongue. I waited two hours; but no amendment was perceptible. I now ordered 8 drops of croton oil to be rubbed into some cerate, and so passed into the rectum. In a few minutes, she complained of great tenesmus and smarting pain in the bowels. Twelve large watery evacuations followed. No more convulsions. I enjoined perfect quiet, and left her.

19th.—Found her better in every respect. No return of convulsions; she complained only of some soreness in the throat; but there was no dyspnœa.

20th.—I was sent for in the night; natural labor was going rapidly on; a large quantity of liq. amnii was discharged, and in a short time she gave birth to a dead child.

21st.—A good night; no pain in the head; lochia natural; urine had been passed; pulse good; tongue moist, but injured during the convulsions. All went on remarkably well till the tenth day, when, as she was sitting up in the bed, drinking a cup of tea, and talking cheerfully with her mother, she suddenly called for a basin; vomited a large quantity of blood, sank back on her pillow, and expired.

The cause of death was evidently from the false teeth, or some portion of the metallic fastening, having penetrated some large vessel. No post-mortem inspection could be obtained.—(*Medical Times and Gazette*, April 23, 1859.)

2. PHYSIOLOGY AND TREATMENT OF PLACENTA PRÆVIA. By *Robert Barnes, M.D.*—The author sought to avail himself of the foundation of the Obstetrical Society of London to elict the experience of its members as to the different principles of treatment of placenta prævia. He submitted fourteen cases whic had come under his own care since the publication of his work on the subject; and appended two series of propositions—the one physiological, the other therapeutical—which appeared to be either proved or illustrated by those cases.

Among the physiological propositions were the following: That in many cases of placental presentation, there arrives a stage when the hemorrhage is spontaneously arrested. That this physiological arrest is not owing to pressure upon the bared surface of the uterus by the bag of liquor amnii, or the child; nor to death of the child; nor to syncope; nor to total detachment of the placenta. That this physiological arrest of the hemorrhage is observed when that part of the placenta which had been implanted within the cervical or lower zone of the uterus has been all detached, contraction of the uterus attending. That, this stage reached, there is no physiological or pathological reason why further detachment of placenta seated within the middle and fundal zones should occur until after the expulsion of the child, when, and not till then, the remainder of the placenta is cast off as in normal labor. That the position of the greater portion of the placenta to the posterior wall of the uterus in these cases, where it forms, by resting on the projecting promontory of the sacrum, a solid inclined plane, directed forward, is a frequent cause of the transverse presentations which are apt to complicate placenta prævia. That in the great majority of cases where an edge of the placenta comes down to the os internum uteri, the umbilical cord springs from this edge, and thus is ready to fall through into the vagina, should the os not be occluded by the child's head.

Among the therapeutical propositions were the following: That owing to the high vascularity and development of the lower segment of the uterus, resulting from this part being the seat of the placenta, uterine inflammation and puerperal fever are exceedingly likely to ensue from the pressure and contusion attending the passage of the child. That this danger is much increased by the forcible introduction of the hand for the purpose of turning and extracting the child before the os uteri has expanded. That in some cases, where it is observed that the placenta has been separated spontaneously from the lower segment of the uterus, the os being expanded to the size of a crown-piece, and the hemorrhage having ceased, it is not necessary to interfere with a labor now become natural *quoad* placental attachment. That since the os internum uteri must expand to the diameter of the child's head, and since, during the dilatation, placenta adhering to the lower segment is liable to successive detachment, causing hemorrhages, it is an indication to expedite this stage of the labor as much as possible. That in some cases the ordinary means of inducing contraction—such as rupturing the membranes, plugging the cervix, ergot or galvanism—will suffice to cause the rapid and safe expansion of the os. That the adhesion of placenta to the lower zone of the uterus impedes the regular progress of labor, and delays the equable expansion of the os uteri. That in those critical cases where forced delivery or the artificial total detachment of the placenta are dangerous or impracticable operations, the introduction of the index finger through the os, and the separation of the part of the placenta adhering to the cervical zone, is a safe and feasible operation.

Dr. Rigby would ask, was Levret's opinion as to the connection between partial implantation of the placenta over the cervix and prolapsus of the funis borne out by Dr. Barnes's experience? He would make one remark on a point which had, he believed, escaped notice. In cases of placenta prævia there now and then occurred danger from slight but continuous hemorrhage after delivery, in consequence of a small rent in the os, which at any other time would be of no consequence, but was of importance in these cases, owing to the extreme vascularity of the cervix.

Mr. Borham had found preternatural position of the child in five out of seven or eight cases of placenta prævia. He was at a loss to explain this. Was it connected with undue shortness of the cord?

Dr. Elkington believed no universal law could be laid down for treatment of these cases. When the placenta was partially implanted over the cervix, and the position of the child natural, he believed that rupture of the membranes and the use of ergot were sufficient. If the implantation was complete, the patient exhausted from loss of blood, etc., the proper treatment was to turn. His success had been greatest when turning had been performed early. He had always found the os easily dilatable when much hemorrhage had occurred, even though only as large as half a crown. He could not consider that the patient was secure from further hemorrhage after the placenta had been separated to a certain degree. He would be disposed to deliver as soon as possible.

Dr. Barnes, in reply, stated that in two of the cases of marginal presentation the cord was prolapsed. The cause of the preternatural position of the child in cases of placenta prævia was, as Levret long ago pointed out, the placenta prævia itself. He believed that in cross presentations generally, also, it would be found that the placenta was attached low down, and that this gave rise to the malposition of the child. He had found the os not dilatable in cases where much blood had been lost, although undoubtedly the opposite was the rule. He would submit that the "insecurity" to which Dr. Elkington had referred as hanging over a woman when the hemorrhage had spontaneously ceased, and the consequent necessity for further proceedings, was a feeling having its seat only in the mind of the accoucheur. He hoped to convert Dr. Elkington to a belief in the physiological views enunciated in the paper.—(*Medical Times and Gazette*, April 16, 1859.)

3. ON THE MORE FREQUENT USE OF THE FORCEPS AS A MEANS OF LESSENING BOTH MATERNAL AND FŒTAL MORTALITY. By *Philip H. Harper, F.R.C.S.*—The author first examined the question, What are the ill effects, either to the mother or child, produced by the forceps? and endeavored to show that not one of those usually ascribed to them could properly be attributed to the use of the instrument itself, but only to its abuse. It appeared, from various authorities, that the causes of maternal death after their use were the same as after unassisted tedious labor, and, therefore, that their origin must be sought in the delay, rather than in the use of the instrument; especially so long as it was only applied in the extreme cases of tedious labor. The causes of the large fœtal mortality were likewise to be found in the long-continued and violent efforts made by the uterus on the child previously to its application, and which are more fatal than the compression of the instrument in the proper direction. The cases of unassisted tedious labor, reported by Johnston and Sinclair, pointed to the fact that mere duration alone, without any abnormal circumstance, was a main element in rendering labor dangerous. Both the maternal and the fœtal mortality in their cases was greater in tedious labor than in their forceps cases. The maternal mortality in their craniotomy cases was greater than in either. Having spoken of the general powers of the instruments as extractors and rectifiers, Mr. Harper examined them as compressors, in order to discover how much compression might safely be exercised upon the fœtal head. He mentioned some experiments of his own upon children still-born after footling, and other such cases, where he applied forceps immediately after birth, and, fastening the handles together with India-rubber springs, had left them on for a time, with the effect of much altering the form of the head, and diminishing its diameter, without any apparent injury to the brain. These cases, of course, only bore slightly upon the question of compression previously to the child's death. The brain must be pressed in a direction parallel to the base of its anterior lobes, to produce the dangerous effects spoken of by Radford and others. In practice this was not really so easy as it was to apply the forceps so as to exert pressure upon the prominent parts of the frontal bone anteriorly, and the junction between the middle and lower thirds of the occipital bone posteriorly. When they were applied thus, and compression gradually exerted, the posterior mass of brain was lifted into the hollow of the forceps, while the anterior lobes were depressed; this movement being similar to the one adopted by nature in molding the head into the long oval shape. The author then spoke of the various states which may call for the use of the forceps, dwelling more particularly upon those dependent upon some such peculiarity existing in the uterus itself, such as rheumatism, spasm, irregular action of its fibres, irritability and debility, and which were very frequent causes of lingering and tedious labor. The period of the labor at which they should be applied was a very important question. It was not enough to show a small maternal mortality, but we must also have a small fœtal mortality. The second stage of labor ought to be steadily progressive, and if such be not the case, we ought to interfere. The element of time ought not to be considered. Careful study of the positions assumed by the child's head, while passing through the pelvis, and not subjected to forcing pains without progressing, showed that the earlier the forceps were applied, the more favorable was the position in which they compressed the head. The mother was unexhausted, flooding was avoided, the soft parts were uninjured, and the child was alive. In examining the various states in which their early use is advisable, the author dwelt especially upon inertia and sluggishness of the uterus. To rouse an overworked and overtasked organ to fresh exertion, was a very questionable proceeding. He brought forward statistics to show that ergot exerted a most deleterious action upon the fœtus. He had long ceased giving it under any circumstances previous to the birth of the child, but always used the short forceps instead, and with very great advantage both to mother and child. In cases of disproportion, which for any reason did not admit of turning, the forceps should be applied early, especially as there was nothing more dangerous than the head being impacted in any one position. In bringing forward various statistical tables to prove the proposition, that "the earlier and more frequently the forceps are applied in proper cases, the more maternal and fœtal lives are saved," he separated all arm, breech, footling, and placental presentations, together with their maternal and fœtal mortality. It was necessary, also, to separate puerperal fever cases and those in which death arose from other labor causes, or from constitutional causes coincident with the occurrence of labor. The necessity for thus dealing with the statistics, in order to arrive at a just conclusion, prevented his using all the obstetric histories which had been published, as they did not all contain these data. He examined Collins, Hardy, and McClintock, Johnston, and Sinclair, and his own statistics, and from them considered the proposition confirmed and proved.—(*British Medical Journal*, June 25, 1859.)

